# LB07 Role of Psychiatric disorders in association of headache and temporomandibular disorders

**DOI:** 10.1186/1129-2377-1-S14-P223

**Published:** 2013-02-21

**Authors:** M Nazeri, F Abareghi

**Affiliations:** 1 Medical Students Research Committee, Medical Sciences University, Kerman, Iran

## Introduction

Temporomandibular Joint Disorders(TMD) is a heterogeneous group of symptoms with a multifactorial etiology, affecting 5-7% of general population. Its association with migraine has long been discussed [1], but exact relationship between these two diseases is not clear yet [2]. It is well known that depression and anxiety are common in both TMD and HA patients, so we hypothesized that headache patients with anxiety and depression are more prone to develop TMD and designed a case-control study to test this.

## Material and methods

In this case-control study, cases consisted of 65 migraine patients(13 Male,52 Female)referring to Neurology clinic and 52 healthy controls(14 Male,38 Female) referring to dental school were group matched with them. Migraine patients were classified according to 2nd edition of International Classification for Headache Disorders(ICHD-II). We examined signs and symptoms of TMD using RDC/TMD(Research Diagnostic Criteria for Temporomandibular Disorders) and HADS-14(Hospital Anxiety and depression Scale) was used to screen anxiety and depression, stratification was performed on the basis of depression and anxiety with cut off score of 7, and scores more than 7 were considered anxious and/or depressed. Data were analyzed by chi-square test with a significance level of 5% and Odds Ratio(OR)test with a 95% Confidence Interval(CI).

## Results

In anxious group, migraine patients had a seven time risk of developing TMD (95% CI=2.4-19.1,p<0.001) while in non-anxious group, such a relationship was not found between headache and TMD(chi-square test, p>0.05). In depressed group, OR for TMD in migraine patients in contrast to controls was 8.5(95%CI=2.5-28,p<0.001), this relationship was also true for non-depressed group with the OR of 3.6(95%CI=1.1-11.6,p<0.001).

## Conclusion

Migraine patients with high level of anxiety seem to have a bigger chance of developing TMD, while high level of depression doesn’t seem to have such an effect. Anxiety should be considered while treating migraine patients with signs of TMD.
